# Relative Importance of Biotic and Abiotic Forces on the Composition and Dynamics of a Soft-Sediment Intertidal Community

**DOI:** 10.1371/journal.pone.0147098

**Published:** 2016-01-20

**Authors:** Travis G. Gerwing, David Drolet, Diana J. Hamilton, Myriam A. Barbeau

**Affiliations:** 1 Department of Biology, University of New Brunswick, Fredericton, New Brunswick, E3B 5A3, Canada; 2 Department of Biology, Mount Allison University, Sackville, New Brunswick, E4L 1G7, Canada; Consiglio Nazionale delle Ricerche (CNR), ITALY

## Abstract

Top-down, bottom-up, middle-out and abiotic factors are usually viewed as main forces structuring biological communities, although assessment of their relative importance, in a single study, is rarely done. We quantified, using multivariate methods, associations between abiotic and biotic (top-down, bottom-up and middle-out) variables and infaunal population/community variation on intertidal mudflats in the Bay of Fundy, Canada, over two years. Our analysis indicated that spatial structural factors like site and plot accounted for most of the community and population variation. Although we observed a significant relationship between the community/populations and the biotic and abiotic variables, most were of minor importance relative to the structural factors. We suggest that community and population structure were relatively uncoupled from the structuring influences of biotic and abiotic factors in this system because of high concentrations of resources that sustain high densities of infauna and limit exploitative competition. Furthermore, we hypothesize that the infaunal community primarily reflects stochastic spatial events, namely a “first come, first served” process.

## Introduction

Ecologists have long debated factors that structure biological communities [1–4]. Abiotic factors, such as salinity or temperature, coupled with variations in tolerance or preference organisms exhibit for these factors [5–7], exert an obvious influence on biological communities [6, 8, 9]. Biotic factors can also affect community composition and spatiotemporal dynamics. Some communities are controlled via predation in a top-down manner [10–12], while others are driven by availability of resources in a bottom-up manner [13–16]. In reality, most communities are likely influenced by a combination of top-down and bottom-up forces [17–20]. Further complicating matters is the role of middle-out variables, such as mid-trophic level predators, often referred to as mesopredators [21]. These animals, frequently omnivores [22, 23], can exert a strong structuring pressure upon biological communities [17, 24, 25]. Identifying the occurrence of each of these processes in a community is relatively straightforward; however, it is more difficult to quantify the relative importance of these processes, occurring concurrently, in determining community structure and dynamics [26–30].

The intertidal mudflats in the Bay of Fundy, Canada, exhibit a moderately complex community [31–33], and provide an ideal system in which to investigate the relative contribution of biotic and abiotic factors to community and population variation. This community appears to be structured by a combination of top-down and bottom-up forces [31, 34, 35]. Potential bottom-up forces include highly productive populations of benthic diatoms, which form the base of this food web [33, 36, 37]. Diatom production is supplemented by high inputs of detrital organic matter [33, 38], likely from local saltmarshes [39]. Potential top-down forces include epibenthic predators such as benthic fish [40], the mudsnail *Nassarius obsoletus* (previously *Ilyanassa obsoleta*; [41]), and shorebirds [31, 34]. In addition, infaunal polychaete omnivores such as Phyllodocidae, Nereididae, and Nephtyidae [33, 42–44] may represent strong middle-out forces [22, 23, 45]. Finally, abiotic factors such as particle size of sediments [46], exposure time to air [47], and dissolved oxygen content in sediments [9] may also be exerting structuring influences.

The goal of our study was to quantify the relative contribution of biotic (top-down, middle-out, bottom-up) and abiotic factors to community and population patterns for an intertidal soft-sediment system. We intensively sampled biotic and abiotic variables of 8 mudflats spanning the entire upper Bay of Fundy over two years [33], and used a multivariate empirical modelling method (PRIMER; [48]) to relate independent variables to the biological community. A notable aspect of our methodology is that it incorporates our spatial and temporal sampling structure (which we termed structural factors), and enables us to disentangle and evaluate the importance of these structural factors and various covariates (biotic and abiotic variables). As such, our study contributes to the understanding of patterns in the mudflat community, and complements earlier manipulative studies focussed on processes occurring over limited spatiotemporal scales [49–51].

## Materials and Methods

### Study Sites

Our 8 intertidal mudflats (termed “sites”) in the Bay of Fundy, Atlantic Canada, consisted of Mary’s Point (MP: latitude 45.72°, longitude -64.67°), Daniels Flats (DF: 45.79°, -64.61°), Grande Anse (GA: 45.82°, -64.50°), Pecks Cove (PC: 45.75°, -64.49°) and Minudie (MN: 45.77°, -64.38°) located in Chignecto Bay, and Moose Cove (MC: 45.29°, -63.81°), Avonport (AV: 45.11°, -64.24°) and Starrs Point (SP: 45.12°,-64.37°) located in Minas Basin (S1 Fig). Details of the biotic and abiotic characteristics of these sites can be found in Gerwing et al. [33], Gerwing et al. [52], and Bringloe et al. [53]. An animal care permit was not required for this study, because we sampled common invertebrates (according to University of New Brunswick guidelines) in publically owned and non-protected areas (intertidal mudflats located below the low high tide line, according to Canadian guidelines).

### Mudflat Sampling

#### Biota

Over two years, 2009–2011, we sampled mudflats every 3 weeks from June to August, and every 6–8 weeks after the end of August until May. Sampling rounds (periods of 5 days when all the mudflats were sampled [42], termed “Round” in our models) occurred at approximately the same time each year (±1 week). For stratified random sampling at each mudflat, we established two transects perpendicular to the low waterline, each divided into 4 equal zones based upon intertidal distance across-shore (this effectively represented 8 strata per mudflat for the analysis in the present paper). More details of the sampling scheme can be found in Gerwing et al. [33]; see also S1 Table.

For mudflat infauna, we randomly selected one sampling location (1 m^2^; hereafter termed “plot”) per zone, for a total of 8 plots per site and an overall total of 1021 plots (data from 3 plots were not used because of missing data). Note that we actually sampled the biota (but not the abiotic variables; see below) at 3 randomly selected plots per zone per transect [33]; preliminary analysis (see S1 Table) indicated that population and community patterns were similar when the dataset was reduced to one plot per zone. Hence, we used the subset of our data in which each plot contained all biotic and abiotic measurements. At each plot, a 7-cm diameter corer was pushed into the sediment as deep as possible (5–10 cm; until hard bottom or the end of the corer was reached). Within 12 h of collection, samples were passed through a 250-μm sieve [54] to retain all life stages of benthic macrofauna, as well as large meiofauna, and preserved in 95% ethanol. We quantified densities of *Corophium volutator*, *Macoma* spp., Copepoda (identified to subclass; mostly from the order Harpacticoida), Ostracoda (identified to class) and Polychaetes (identified to family; as in Gerwing et al. [33]).

For each plot, we determined concentration of chlorophyll *a*, an indicator of diatom abundance, in the top 2–3 mm of the sediment, as in Coulthard and Hamilton [55]. We estimated the proportion of the plot covered in shorebird footprints, which were generated primarily by Semipalmated Sandpipers (*Calidris pusilla*), the most abundant shorebird species in this area [56]. This is a good indication of sandpiper habitat use [57], and of foraging activity within a plot since sandpipers spend the majority of their time foraging while on the mudflats [58]. We counted the numbers of *N*. *obsoletus* snails and fish feeding traces (hereafter termed “fish bites”) in each plot (see Risk and Craig [59] and McCurdy et al. [40] for images of fish bites and identification criteria).

### Abiotic Variables and Sediment Properties

Transects extended from the landward start of the mudflat (immediately after the narrow pebble/sandy beach or salt marsh) to the highest low water line, and were 700–1800 m long, depending on the across-shore size of the mudflat. We calculated an index of exposure time (time out of water) for each plot as:

1–[plot distance(m)from shore divided by total transect distance(m)].

This index of air exposure is adequate, because the elevations of the start and end of our transects were similar among mudflats (based on tide dynamics), and the angle of repose (slope) of these expansive mudflats appears generally consistent. In each plot, we evaluated penetrability of sediment by dropping a metal rod (15 cm long, 1.9 cm diameter, 330 g) from 0.75 m above the substratum (i.e., distance from bottom of the rod to the top of the sediment). The depth (mm) that the rod penetrated into the sediment was recorded [60]. We measured depth of the apparent redox potential discontinuity (aRPD), an index of the general sediment dissolved oxygen content [61], to the nearest 0.5 cm in the void left in the sediment following removal of the 7-cm diameter core for infaunal sampling [52]. We determined additional sediment properties by collecting one sediment sample (corer: 3-cm diameter, 5-cm deep) from each plot, and quantified organic matter content, water content and volume-weighted mean particle size in the top 1 cm of the sediment, as in Gerwing et al. [33].

### Data Analysis

#### Environmental Factors Associated with Community Structure

All data analyses were conducted using the statistical program PRIMER with the PERMANOVA (Permutational Multivariate Analysis of Variance) add-on [62]. We used a PERMANCOVA, a multivariate analysis of covariance, to determine which of our covariates (Abiotic: air exposure index, mean particle size, water content, sediment penetrability, aRPD depth; Biotic top-down: percent cover of sandpiper footprints, density of *N*. *obsoletus*, density of fish bite; Biotic bottom-up: chlorophyll *a* concentration, organic matter content) were associated with the spatiotemporal variation of the infaunal community. Prior to analysis, we assessed possible correlations between all pairs of covariates by calculating univariate Pearson’s correlation coefficients. We used a threshold of 0.95 [63] to determine if variables were too correlated to be considered independent. As the highest correlation coefficient observed was 0.86, all variables were included in our models. As part of the PERMANCOVA, we quantified variance components, the proportion of the multivariate variation accounted for by each variable [64, 65]. The infaunal community included *Macoma* spp., *C*. *volutator*, Copepoda, Ostracoda, and polychaetes (Capitellidae, Spionidae, Cirratulidae, Nereididae, Nephtyidae, and Phyllodocidae). A resemblance matrix of the infaunal densities was calculated using Bray-Curtis coefficients, and a dummy variable of 1 to deal with plots with no infauna [66]. Taxa densities were fourth root transformed to improve assessment of rare and common taxa on community structure [48]. All covariates were normalized prior to analysis to handle measurements with different units and scales (e.g., μm, number m^-2^). Mean particle size, chlorophyll *a* concentration, density of fish bites, and density of *N*. *obsoletus* were fourth root transformed prior to normalization to correct skewed distributions [63]. Middle-out polychaetes (Phyllodocidae, Nereididae, and Nephtyidae) were omitted as covariates in this infaunal community analysis since they were part of that community. Beyond the covariates, Round (8 levels per year) was included as a fixed factor, while Year (2 levels) and Site (8 levels) were included as random factors. Year, Round, Site, and Plot (i.e., the lowest level of replication) are hereafter referred to as structural factors. We used α = 0.05 for the community analysis, and tested homogeneity of slopes by examining the interaction between structural variables and covariates. Non-significant interactions with covariates were removed from the model, and significant interactions with covariates were interpreted as contributing to the proportion of the community variation accounted for by the involved covariate [65]. Since we used Type I sums of squares and our dataset was mildly unbalanced (data from only 3 plots were missing), we repeated the PERMANCOVA with the various independent variables entered in different orders and verified that variable order within the model did not alter results [48, 65]. Finally, covariates that did not account for any variation in the multivariate data cloud were removed [67].

### Environmental Factors Associated with Individual Taxa

To evaluate the variables associated with population densities of individual taxa, a resemblance matrix was constructed for each taxon (density data fourth-root transformed, Brays-Curtis coefficient, and a dummy variable). We used the same type of analysis as for the community data to ease comparison between taxa patterns and community patterns. Note that PERMANOVA/PERMANCOVA are appropriate for univariate analysis [48, 65]. We used the same covariates as detailed for the community analysis, and we added middle-out polychaetes as covariates for the taxon-specific analyses. Phyllodocidae, Nereididae, and Nephtyidae were fourth-root transformed prior to normalization when used as covariates. We conducted PERMANCOVAs as detailed above, and repeated them to test for the possible effect of order of independent variables; variable order only affected the statistical results for one taxon (Nephtyidae), but did not change the general interpretation for that taxon. To correct for possible inflation of family-wise error rates in these multiple taxon-specific analyses, we used α = 0.01 [8]. We calculated Pearson’s correlation coefficient between the density of each taxon and each of its significant covariates to assess the nature of the pattern.

## Results

### Environmental Factors Associated with Community Structure

Structural factors accounted for the majority of the observed infaunal community variation (~79%; Table 1, S2 Fig and S3 Fig). Spatial factors (plots 37% and sites 32%) accounted for most of this variation, while temporal factors (year and round) accounted for a significant, but small proportion of the variation. Bottom-up factors also contributed significantly to community variation, although chlorophyll *a* concentration only accounted for ~1% of the variation. Top-down factors accounted for ~6% of the variation. Of the top-down predators, *N*. *obsoletus* (and interactions involving *N*. *obsoletus*) accounted for the largest proportion of the variation (4.7%), while sandpipers (0.4%) and fish bites (1.1%) accounted for a minority of the variation (Table 1). Abiotic covariates accounted for 11% of the community variation. Our index for air exposure (and interactions involving it) accounted for the most (~9%), while mean particle size (and interactions involving it) accounted for a small proportion (~2%) of the variation.

**Table 1:** Results of PERMANCOVA determining which of the covariates and structural factors were associated with the infaunal community change over space and time for Bay of Fundy mudflats in 2009–2011. We used 996–999 unique permutations. Significant *p* values are in bold. Only interactions between structural factors and covariates that were significant are presented.

Variable Type	Source	df	MS	Pseudo-F	*p*	Variance component (%)
Abiotic	**Exposure**	1	68215	98.35	**0.001**	**4.37**
	**Exposure x Round**	7	1651	2.91	**0.001**	**0.57**
	**Exposure x Site**	7	7541	13.49	**0.001**	**3.87**
	Particle Size	1	18872	1.21	0.358	0.23
	**Particle Size x Round**	7	2250	2.83	**0.001**	**0.85**
	**Particle Size x Site**	7	1477	2.46	**0.001**	**1.10**
	Penetrability	1	11842	1.44	0.241	0.38
	aRPD Depth	1	8884	1.43	0.264	0.23
	**aRPD Depth x Site**	7	1041	1.57	**0.036**	**0.32**
Biotic: Top-down	**Sandpiper Footprints**	1	8468	4.28	**0.002**	**0.42**
	***N*. *obsoletus***	1	68222	9.04	**0.001**	**4.30**
	***N*. *obsoletus* x Site**	7	1053	1.82	**0.005**	**0.35**
	**Fish Bites**	1	5071	3.10	**0.02**	**0.24**
	**Fish Bites x Year**	1	2090	3.52	**0.003**	**0.34**
	**Fish Bites x Site**	7	1533	2.35	**0.001**	**0.53**
Biotic: Bottom-up	Organic Matter	1	33715	1.41	0.277	1.24
	**Chlorophyll *a***	1	23870	3.06	**0.017**	**1.09**
Structural	**Year**	1	6879	2.55	**0.077**	**0.68**
	**Round**	7	10280	3.60	**0.001**	**4.68**
	**Site**	7	52501	31.44	**0.001**	**31.59**
	Year x Round	7	1253	1.32	0.147	0.39
	**Year x Site**	7	1484	2.67	**0.001**	**1.29**
	Round x Site	49	1048	1.14	0.178	0.65
	**Year x Round x Site**	49	926	1.67	**0.001**	**3.58**
	**Residual** (a.k.a. Plot)	834	556			**36.71**
	Total	1020				

### Environmental Factors Associated with Individual Taxa

Similar to the community analysis, structural factors (particularly spatial factors) accounted for the majority of the variation in taxon-specific analyses (Table 2). Abiotic, bottom-up, middle-out, and top-down covariates accounted for a smaller proportion of the variation; however, the pattern of significant variables and the proportion of the variation they accounted for varied among taxa, and with the community analysis. Middle-out covariates were associated with many of our taxa, and they accounted for a relatively large amount of the variation, especially for sessile polychaetes (Capitellidae, Spionidae, and Cirratulidae; 12–21%).

**Table 2:** Summary results of the PERMANCOVAs determining which covariates were associated with each taxon’s spatiotemporal variation for Bay of Fundy mudflats in 2009–2011. Values represent the percent of the variation accounted for by each independent variable (i.e., variance components). The sign in parenthesis represents the nature (+/-) of the Pearson’s correlation coefficient between the response taxon’s density and that variable. Empty cells represent independent variables which did not account for any of the variation (in this situation, covariates were removed from the models, and structural factors were pooled).

Variable Type	Variable	*C*. *volutator*	Ostracoda	Copepoda	*Macoma* spp.	Capitellidae	Spionidae	Cirratulidae	Phyllodocidae	Nereididae	Nephtyidae
Abiotic	Exposure	0.28 (-)**	1.89 (+)***		12.55 (+)***	0.38 (+)***	4.36 (-)***	0.65 (+)***	0.40 (+)*	4.48 (+)***	12.18 (-)***
	Exposure x Round	1.45***							1.68***		
	Exposure x Site	4.41***	0.99**		4.04***	3.64***	3.44***	5.25***		0.03***	4.79***
	Particle Size			2.43 (-)**							
	Particle Size x Site										2.22***
	Water Content						3.17 (-)**				
	Water Content x Site										2.79***
	Penetrability		2.75 (+)*								
	aRPD Depth	1.92 (-)**		0.59 (+)*							
	aRPD Depth x Site	1.95***									
Biotic: Top-down	Sandpiper Footprints	0.98 (+)***								0.56 (-)*	
	*N*. *obsoletus*		2.04 (+)*			3.25 (+)**			2.77 (+)***		
	*N*. *obsoletus* x Site		0.83**				1.47***				1.09***
	Fish Bites	0.33 (+)*			0.21 (-)*						
	Fish Bites x Year						0.71**				
	Fish Bites x Site	2.13***									
Biotic: Middle-out	Nereididae	4.13 (+)***	6.11 (-)*		6.81 (+)***	5.02 (-)**	0.97 (-)*			na	
	Nereididae x Site			1.43***				2.09***		na	
	Nephtyidae			3.26 (-)*			0.51 (+)*				na
	Nephtyidae x Site					3.00***		4.05***			na
	Phyllodocidae		3.92 (+)*	3.79 (+)***		9.18 (+)**	9.82 (+)***	13.44 (+)***	na		
	Phyllodocidae x Site	2.28***		1.08**			1.19***	1.39***	na		
Biotic: Bottom-up	Organic Matter			4.82 (+)**			2.71 (-)*				
	Organic x Round	0.99**									
	Chlorophyll *a*	1.15 (-)*				4.32 (+)**		3.62 (+)**			
Structural	Year			1.41*							
	Round	6.29***	1.06*		2.19**			3.44***	2.15**	5.82***	
	Site	17.27***	29.49***	11.49***	9.72***	28.51***	5.37*	24.83***	12.01***	23.02***	23.47***
	Year x Round			1.75*							
	Year x Site		1.09**	0.89*			1.95**				
	Round x Site	4.29***			3.11***		7.84***		2.49*	2.23***	
	Year x Round x Site			5.85***							
	Residual	50.09	45.30	58.97	59.05	41.59	54.45	36.58	76.24	61.31	52.38

na = not appropriate; for the analysis of Phyllodocidae, Nereididae or Nephtyidae, the middle-out covariate for that taxon was not used. We used 996–999 unique permutations.

*, ** and *** represent *p* ≤ 0.05, *p* ≤ 0.01 and *p* ≤ 0.001, respectively; however, only terms with ** and *** were interpreted.

## Discussion

### Relative Contribution of Biotic, Abiotic, and Structural Factors to Mudflat Community Structure

Structural factors (those related to space) were responsible for the majority of observed variation in the infaunal community of the Bay of Fundy mudflats. Although, and as in other systems, we observed that the community also varied significantly with top-down [10, 11], bottom-up [14, 20], and abiotic variables [6, 8]; however, these explained relatively little of the overall variability in the system. Previous experimental studies in the Bay of Fundy have found that both top-down and bottom-up forces influenced mudflat communities [31, 34]. Our results do not disagree with these findings, but suggest that on a broad scale the importance of those factors is limited. These past studies were conducted on a smaller spatiotemporal scale than ours, and so only tested the effects of these factors on spatially and temporally localized processes. Two strengths of our study are the broad spatiotemporal scale, and the multitude of factors examined concomitantly, both of which allow us to investigate the relationship between these factors and patterns in community structure. The limited importance of top-down and bottom-up variables in our study suggests that infaunal community structure may be relatively decoupled from both top-down and bottom-up factors in this type of habitat. Instead, we suggest that structural factors, which accounted for approximately 79% of community variation, may reflect stochastic events; for example, temporal factors may be related to interactions between time of year (seasons: temperature, photoperiod) and weather patterns [68, 69]. The influence of the spatial factor Site (at the scale of kilometres) may be related, in part, to processes such as larval supply [70], post-settlement dispersal [71], unmeasured site features (e.g. hydrodynamic patterns or shelter from tides/waves; [72]), or their interaction. Sediment type (particle size), typically an important site-level feature in soft-sediment studies [4, 6, 73, 74], would not have been greatly influential in our study because we had a small range of sediment types among our silt-dominated mudflats [53]. The variation among plots (at the spatial scale of tens to hundreds of meters) may be a result of fine-scale interactions such as local hydrology and delivery of larvae to a plot, intra- and interspecific interactions among infauna, and/or post-settlement dispersal [41, 71, 73–76]. Overall, the mudflat infaunal community may reflect a “first come, first served” situation, as described in community succession models [77], and discussed further below.

Community dynamics that are uncoupled from top-down predation (i.e., where predation, even when common, has a minor influence on measured phenomenon) have been observed before in resource-pulse ecosystems. In these situations, resource-driven increases in prey numbers are so large that predators exert little influence upon density of prey species [17, 20, 78]. We propose that the annual bloom in benthic diatoms observed during spring/summer in our system [33, 37] acts as a resource pulse, resulting in such an increase in infaunal density [33] that top-down predation has little lasting effect. Further, while predators such as shorebirds may have substantial short-term impacts on certain taxa, the mortality is likely compensatory in nature (sensu [79]). Seasonal declines in many invertebrates occur regularly in this region [80], so predators are consuming soon-to-die individuals. Indeed, Hamilton et al. [31] suggested that although foraging by sandpipers coincided with large declines in *Corophium volutator*, much of this mortality would have occurred anyway.

Although we propose that top-down effects in our mudflat system were largely neutralized by a superabundance of resources, it should be noted that benthic chlorophyll *a* concentration (a measure of diatom abundance) accounted for only a small proportion of the infaunal community variation and sediment organic matter content was not significant. Thus, community structure and dynamics may also be relatively uncoupled from bottom-up factors. Ample available resources should not necessarily be interpreted as bottom-up control of a system, at least not in the sense that resources tightly influence community structure and dynamics (as in resource-limited systems). The high primary productivity on mudflats during spring and summer [33, 37] likely limits the importance of exploitation competition, as in resource-pulse ecosystems [17, 20, 78]. Indeed, Drolet et al. [41] found no evidence of intraspecific competition among the highly abundant *C*. *volutator* in our mudflat system, and attributed this to the presence of ample resources. The high amount of resources observed for the majority of the year on our mudflats may be above the threshold required to sustain infaunal populations, and so would minimize the impact of predation as well as limit exploitative competition.

Strong relationships between abiotic factors and community/population densities have been documented in previous studies [4, 6, 46, 73, 74]. However in our study, measured abiotic variables (air exposure index, and mean particle size, penetrability, water content and oxygen content of sediment) accounted for just 11% of the variation in the infaunal community. Of the abiotic variables we examined, air exposure (i.e., index of time emersed) accounted for the largest proportion of the community variation (~9%). This suggested that the infaunal community exhibited subtle across-shore zonation, an observation previously reported for *Macoma balthica* on Bay of Fundy mudflats [47]. Zonation is likely a result of differential exposure tolerance of organisms [5] and is common but subtle on intertidal mudflats [81–83]. While abiotic variables accounted for more of the community variation than biotic variables, both accounted for less than structural factors. The relatively low importance of abiotic variables may be partially related to the limited variability in conditions observed among our mudflats. In addition, high resource concentrations, as in resource-pulse ecosystems, can lower the influence of abiotic factors by attracting animals to habitats or patches with abiotic characteristics that would normally preclude occupancy [84, 85]. This may be occurring in our system, because we have observed high densities of *C*. *volutator* in sandy-mud patches rich in chlorophyll *a* [33], despite this animal’s tendency to avoid areas of relatively coarse sediments [46, 86, 87]. Indeed, Meadows [88] observed that *C*. *volutator* can settle, after a swimming event, on sandy sediments, but avoided settling there if sand was treated to remove biofilm.

### “First-Come, First Served” Hypothesis as a Structuring Force on Intertidal Mudflats

In sum, intertidal mudflats have high primary productivity [37, 83, 89], as well as muted temperature, desiccation and salinity stresses compared to other intertidal habitats [83, 90]. Mudflats have less competition for space than rocky shores or salt marshes, given the three-dimensional aspect of the substrate [83, 90–92], have a low angle of repose, and are often expansive, which contributes to diffuse predation pressure by mobile predators [22, 34, 83, 90]. Therefore, mudflats may be viewed as a relatively benign environment for organisms adapted to living in mud. We hypothesize that mudflat community structure and dynamics are mostly not a result of the above-mentioned factors, but are instead mainly reflective of a “first come, first served” process [77, 83, 93]. Initially, delivery of larvae (for species with a dispersive larval phase) and movement by juveniles and adults [53, 71, 94] may be important at the spatial scale of sites (which would help explain the large proportion of variation that this term accounted for in our analysis). Recent work by Pilditch et al. [71] identified dispersal by juveniles and adults (i.e., post-settlement dispersal), which is characterized by continued, frequent, small-scale movements over long periods, as being key to the dynamics of soft-sediment communities. This may be occurring in the Bay of Fundy, as *C*. *volutator* juveniles and adults have been observed to disperse substantially both between and within mudflats [53, 94, 95]. We hypothesize that, once established, residents may then resist colonization by dispersing individuals [96, 97], leading to a first-come first-served situation for community structure. In future studies, we are interested in formally testing whether pre-emptive competition may be an important structuring force of mudflat infaunal communities, contributing to first-come, first-served dynamics.

### Patterns at the Taxon Level, and Assessment of Middle-out Forces

As in our community analysis, the majority of the spatiotemporal variation of each taxon was accounted for by structural factors (Table 2). Furthermore, and similar to other systems, most taxa varied with a combination of abiotic [6], top-down [11], bottom-up [20], and middle-out variables [21, 24, 25]. However, not only was there a different pattern in the importance of independent variables among taxa, but also between taxa and the community as a whole (Tables 1 and 2). For instance, the community level analysis suggested that sediment water and organic matter content were not associated with community variation. However, taxon-specific analyses revealed that water content was associated with Spionidae densities, and organic matter with copepod densities.

Generally, middle-out polychaetes accounted for a relatively large proportion of population variation when compared to top-down and bottom-up variables (Table 2). The association between sessile infauna (Capitellidae, Spionidae, and Cirratulidae) and mesopredators was often relatively high, likely because sessile animals cannot easily avoid predation [22] or bioturbation [98]. However, the influence of middle-out predators was still limited compared to structural factors, perhaps as a result of low mesopredators densities [33]. Nevertheless, even if mesopredator density had been higher, intraguild predation would likely have resulted in limited suppression of prey species [99], since these polychaetes are omnivores [42–44].

### Implications of Different Results for Community and Taxon-Specific Analyses

The methods used in our paper can be applied to any system to quantify the relative importance of various potential structuring forces. In our study, the variation in patterns reported between taxa, even taxa performing similar ecological roles, for example Cirratulidae and Spionidae [42–44], suggests that generalizations cannot always be made. Murray et al. [100] arrived at a similar conclusion when they observed that species sharing traits cannot always be aggregated into the same functional group. Although empirically modelling the community as a whole offers a useful method to understand community spatiotemporal dynamics, it may obscure important associations between individual taxa and structuring variables. Indeed, Spasojevic and Suding [101], in their examination of plant community functional diversity, observed that multivariate analyses obscured key relationships which were subsequently identified by analysing individual traits (abiotic filtering, above-ground competition, etc.). Continued work is required to evaluate the limitations of community models that overlook less common taxa due to the influence of more common taxa. In many situations, less common taxa may exert such a minor influence on the community that the community approach is appropriate. In other situations, community-level models that obscure factors influencing key but less common taxa, such as ecosystem engineers [102, 103], may fail to quantify essential interactions. It is likely that the answer to this question varies among biological systems and the features of the system investigated.

### Conclusions

Previous studies in Bay of Fundy intertidal mudflats reported significant effects of top-down, bottom-up, and abiotic factors on infaunal dynamics [31, 34]. Manipulative experiments such as these are instrumental in identifying processes that operate within an ecosystem, but are less efficient at quantifying how these processes interact to produce patterns at larger scales. This is because manipulative experiments are logistically constrained to a limited number of variables [104], and cannot manipulate the full suite of *in situ* conditions [11, 32, 34, 84, 92]. Although correlational, broad spatiotemporal sampling and statistical analyses such as presented in our study help to reveal patterns and the relative contribution of different structuring processes within a system. For our soft-sediment intertidal community, we found that the majority of the observed spatiotemporal variation in infauna was accounted for by structural factors (site, plot), rather than measured top-down, middle-out, bottom-up and abiotic variables. This may be a result of high concentrations of resources, as in resource-pulse ecosystems [17, 20, 78]. Based on our results and known features of mudflats, we now hypothesize that the infaunal mudflat community primarily reflects stochastic events, namely an assemblage of taxa that first recruit onto the mudflat (a “first-come, first-served” model).

## Supporting Information

S1 Fig Map of study sites (i.e., intertidal mudflats) in the Bay of Fundy, Eastern Canada. (DOCX)

S2 Fig Non-metric multidimensional scaling (nMDS) plots of the infaunal community composition on eight intertidal mudflats (a.k.a. sites) in the upper Bay of Fundy, Canada, and eight sampling rounds per year over two years (2009–2011). (DOCX)

S3 Fig Bubble plots of the nMDS plots from S2 Fig, representing the magnitude of covariates (abiotic, top-down or bottom-up variables) that accounted for the highest proportion of the observed variation in the infaunal community. (DOCX)

S1 Table Preliminary PERMANOVAs conducted to determine if the smaller community dataset (n = 4 samples per transect dataset), which contained all biotic and abiotic variables, produced similar results to the full community dataset (n = 12 samples per transect), which did not contain all abiotic variables. (DOCX)
