# Supplementary material for: Relative Importance of Biotic and Abiotic Forces on the Composition and Dynamics of a Soft-Sediment Intertidal Community
Source: PLoS One. 2016 Jan 20;11(1):e0147098. doi: 10.1371/journal.pone.0147098 (PMC4720360; doi:10.1371/journal.pone.0147098)
Supplement: S1 Fig — (DOCX) [file pone.0147098.s001.docx]

**
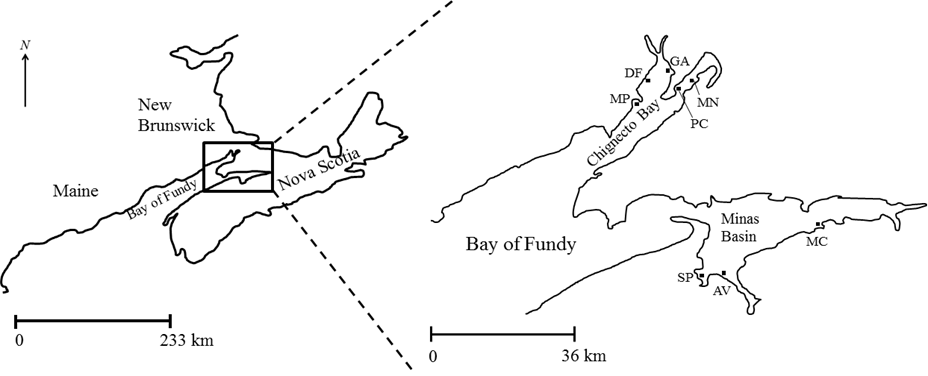
**

S1 Fig: Study sites (i.e., intertidal mudflats) in the Bay of Fundy, Eastern Canada. Site names are Starrs Point (SP), Avonport (AV), Moose Cove (MC), Minudie (MN), Pecks Cove (PC), Grande Anse (GA), Daniels Flats (DF), and Mary’s Point (MP).
