# Supplementary material for: Relative Importance of Biotic and Abiotic Forces on the Composition and Dynamics of a Soft-Sediment Intertidal Community
Source: PLoS One. 2016 Jan 20;11(1):e0147098. doi: 10.1371/journal.pone.0147098 (PMC4720360; doi:10.1371/journal.pone.0147098)
Supplement: S2 Fig — (DOCX) [file pone.0147098.s002.docx]

Phyllodocidae

Cirratulidae

Spionidae

Nereididae

Nephtyidae

Capitellidae

*Macoma* spp.

Copepoda

Ostracoda

*C. volutator*

Phyllodocidae

Cirratulidae

Spionidae

Nereididae

Nephtyidae

Capitellidae

*Macoma* spp.

Copepoda

Ostracoda

*C. volutator*

1

1

1

1

1

1

1

1

2

2

2

2

2

2

2

2

3

3

3

3

3

3

3

3

4

4

4

4

4

4

4

4

5

5

5

5

5

5

5

5

6

6

6

6

6

6

6

6

7

7

7

7

7

7

7

7

8

8

8

8

8

8

8

8

2D Stress: 0.18

Transform: Fourth root

Resemblance: S17 Bray Curtis similarity (+d)

Site

SP

AV

MC

MN

PC

GA

DF

MP

1

1

1

1

1

1

1

1

2

2

2

2

2

2

2

2

3

3

3

3

3

3

3

3

4

4

4

4

4

4

4

4

5

5

5

5

5

5

5

5

6

6

6

6

6

6

6

6

7

7

7

7

7

7

7

7

8

8

8

8

8

8

8

8

2D Stress: 0.16

2009-2010

2010-2011

S2 Fig: Non-metric multidimensional scaling (nMDS) plots of the infaunal community composition on eight intertidal mudflats (a.k.a. sites) in the upper Bay of Fundy, Canada, and eight sampling rounds per year over two years (2009-2011). Each symbol represents an average per combination of site and round. Round 1: early June, 2: late June, 3: mid-July, 4: early August, 5: late August/early September, 6: October, 7: December, 8: March. See S1 Fig for full site names. The stress is < 0.2, indicating that the nMDS plots are adequate 2-dimensional representations of the multidimensional situation. The clustering of sites represents well the strong importance of the spatial structural variable Site in the PERMANCOVA (Table 1 in the paper). The variation among rounds within a site represents the temporal structural variable Round (Table 1). The two plots provide a sense of the variation among years (one of our other temporal structural variables; Table 1). Vector overlays beneath the nMDS plots represent Pearson correlations between taxa and nMDS axes; the vector of each taxon shows the direction of increased density across the nMDS plot.
