# Supplementary material for: Relative Importance of Biotic and Abiotic Forces on the Composition and Dynamics of a Soft-Sediment Intertidal Community
Source: PLoS One. 2016 Jan 20;11(1):e0147098. doi: 10.1371/journal.pone.0147098 (PMC4720360; doi:10.1371/journal.pone.0147098)
Supplement: S3 Fig — (DOCX) [file pone.0147098.s003.docx]

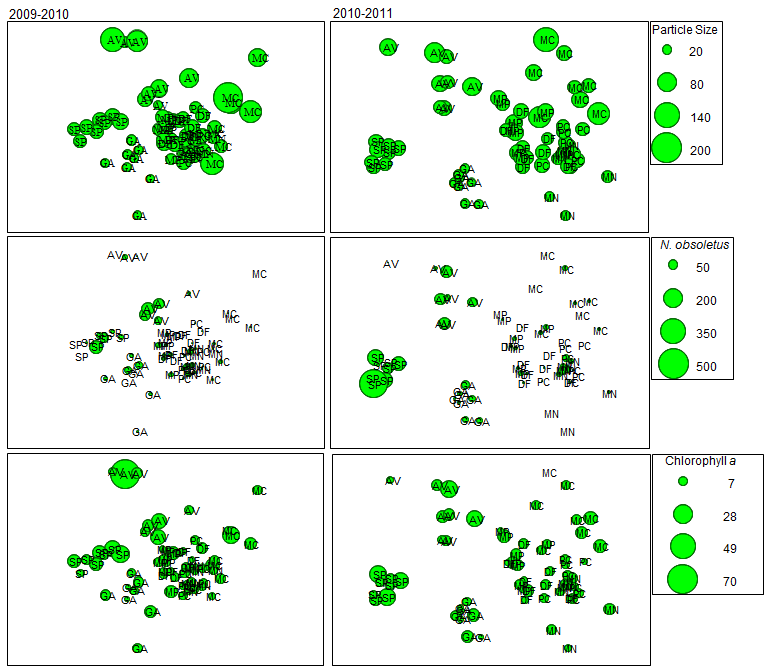


S3 Fig: Bubble plots of the nMDS plots from S2 Fig. Bubble size represents the magnitude of covariates (abiotic, top-down or bottom-up variables) that accounted for the highest proportion of the observed variation in the infaunal community (Table 1 in the paper). Each symbol represents an average per combination of site and round; see S1 Fig for full site names. Units for volume-weighted mean particle size of the sediment in the top 1-cm layer are μm, for density of the snail *Nassarius obsoletus* are number of individuals m^-2^, and for concentration of chlorophyll *a* in the top 2-3 mm of the sediment (a measure of benthic diatom abundance) are mg m^-2^.
