# Supplementary material for: Relative Importance of Biotic and Abiotic Forces on the Composition and Dynamics of a Soft-Sediment Intertidal Community
Source: PLoS One. 2016 Jan 20;11(1):e0147098. doi: 10.1371/journal.pone.0147098 (PMC4720360; doi:10.1371/journal.pone.0147098)
Supplement: S1 Table — (DOCX) [file pone.0147098.s004.docx]

S1 Table: Preliminary PERMANOVAs conducted to determine if the smaller community dataset (a: n = 4 samples per transect), which contained all biotic and abiotic variables, produced similar results to the full community dataset (b: n = 12 cores per transect), which did not contain all abiotic variables. All factors in both analyses are random. Both analyses produced similar results, with Plot and Site accounting for the majority of the variation. Therefore, the smaller dataset, which contains all biotic and abiotic variables, was used for the PERMANCOVA analysis in the main paper. Note that for the full community dataset, we included “Transect” in the analysis, because there were more samples, and some were relatively close to one another (although, always > 20 m). For the smaller community dataset, we did not include “Transect”, because the samples were distant enough from one another (average 270 m), and the transect effect was very small.

| a) n = 4 samples per transect (1 sample per zone) | |  |  |  |  |
| --- | --- | --- | --- | --- | --- |
| Source | df | MS | Pseudo-F | *p* | Variance Components (%) |
| Year | 1 | 7383 | 2.30 | 0.014 | 0.7 |
| Round | 7 | 7902 | 2.78 | 0.001 | 3.2 |
| Site | 7 | 72677 | 24.09 | 0.001 | 39.0 |
| Year X Round | 7 | 1916 | 1.80 | 0.014 | 0.9 |
| Year X Site | 7 | 1757 | 1.65 | 0.034 | 0.8 |
| Round X Site | 49 | 1304 | 1.23 | 0.079 | 1.1 |
| Year X Round X Site | 49 | 1064 | 1.45 | 0.001 | 2.9 |
| Residual | 893 | 732 |  |  | 51.5 |
| Total | 1020 |  |  |  |  |
|  |  |  |  |  |  |
|  |  |  |  |  |  |
| b) n =12 samples per transect (3 samples per zone) | |  |  |  |  |
| Source | df | MS | Pseudo-F | *p* | Variance Components (%) |
| Year | 1 | 24556 | 2.22 | 0.013 | 0.7 |
| Round | 7 | 29767 | 3.10 | 0.001 | 3.8 |
| Site | 7 | 218430 | 13.28 | 0.001 | 36.4 |
| Transect(Site) | 8 | 7067 | 3.57 | 0.001 | 2.0 |
| Year X Round | 7 | 6818 | 3.00 | 0.001 | 1.6 |
| Year X Site | 7 | 5274 | 1.70 | 0.003 | 0.9 |
| Round X Site | 49 | 3521 | 1.42 | 0.001 | 1.9 |
| Year X Transect(Site) | 8 | 1369 | 1.47 | 0.072 | 0.3 |
| Round X Transect(Site) | 56 | 869 | 0.93 | 0.687 | 0.0 |
| Year X Round X Site | 49 | 2273 | 2.44 | 0.001 | 3.8 |
| Year X Round X Transect(Site) | 56 | 931 | 1.34 | 0.001 | 1.3 |
| Residual | 2814 | 697 |  |  | 47.3 |
| Total | 3069 |  |  |  |  |
|  |  |  |  |  |  |
